# Effective removal of naphthalene from water using bacteria and bacteria-load carrier materials

**DOI:** 10.3389/fchem.2025.1597470

**Published:** 2025-06-11

**Authors:** Kui Liu, Wen-Chieh Cheng, Yi-Xin Xie, Lin Wang, Zhong-Fei Xue, Bowen Yang, Hao Zhang, Jia Min, Miao Yao

**Affiliations:** ^1^ China DK Comprehensive Engineering Investigation and Design Research Institute Co., Ltd., Xi’an, China; ^2^ School of Civil Engineering, Xi’an University of Architecture and Technology, Xi’an, China; ^3^ Shaanxi Key Laboratory of Geotechnical and Underground Space Engineering (XAUAT), Xi’an, China

**Keywords:** Naphthalene, degradation efficiency, abiotic loss, carrier material, thermal stability

## Abstract

The volatilization of naphthalene unavoidably poses significant risks to health, the environment, and safety. Traditional remediation approaches have been criticized for their inefficiency in removing naphthalene and transforming its toxicity. This study proposed a bacteria-loaded carrier material and evaluated its degradation efficiency compared to that of free bacteria. High concentrations made it more challenging for *Microbacterium paraoxydans* (ms) to achieve effective degradation of naphthalene. Additionally, the degradation process was not timely, thereby exacerbating the risks associated with the volatilization of naphthalene. Three carrier materials—activated carbon (AC), calcium alginate (CA), and composite gel beads (CO)—were evaluated for their adsorption, biocompatibility, and thermal stability. CO’s adsorption of naphthalene occurred mainly through chemisorption, with π-π conjugation and Ca-π interaction enhancing the adsorption process. The adsorption peaks did not exhibit any shifts after the involvement of bacteria, indicating the best biocompatibility among the carrier materials, despite having the second lowest total weight loss (CA > CO > AC) during the heating process. The salicylic acid pathway and the phthalic acid pathway were involved in the degradation of naphthalene. No signs of naphthalene were seen in the samples from confocal laser scanning microscope (CLSM) tests, indicating that ms fully degraded naphthalene after its adsorption. While ms degraded naphthalene on day 4 for 50 mg/L and 100 mg/L concentrations, 31.2 mg/L remained for the 200 mg/L concentration. In contrast, ms-loaded CO degraded most of the naphthalene on day 1, with only 2.8 mg/L remaining from the initial 200 mg/L concentration. This study underscored the relative merits of applying ms-loaded CO to the degradation of naphthalene.

## 1 Introduction

Naphthalene, due to its low water solubility and high mobility, is considered as dangerous environmental contaminant (Iwegbue et al., 2023; Zhou et al., 2024). Its bioaccumulation poses severe threats to aquatic plants and humans, leading to hemolytic anemia and potential carcinogenicity (Kang et al., 2012; Lewis, 2012; Mallikarachchi et al., 2024). Traditional remediation technologies, such as chemical adsorption, while effective in capturing contaminants, often fail to address the toxicity of naphthalene. These technologies do not remove naphthalene but merely transfer it to another phase, necessitating further treatment (Dai et al., 2022; Dai et al., 2024). Additionally, they are criticized for low degradation efficiency, long treatment times, and high risk of secondary pollution, which severely limit their applicability (Kumar et al., 2021; Gupta et al., 2024; Wang et al., 2024).

Bioremediation has garnered significant attention as a promising alternative to traditional remediation technologies by overcoming existing challenges (Haritash and Kaushik, 2009). This process typically involves the use of degrading bacteria to break down contaminants and has been extensively employed to remove environmental contaminants from air, water, soil in both natural and artificial settings. However, abiotic loss of naphthalene due to its volatilization mean that some of the naphthalene diffuses into the surrounding environment before bioremediation can occur, thereby reducing degradation efficiency (Mozo et al., 2012). Addressing the issue of abiotic loss is therefore crucial to expanding the applications of bioremediation technologies (Xie et al., 2024). In recent years, carrier materials have gained attention because their porous structures with larger specific surface areas provide more adsorption sites (Zhang et al., 2023). This characteristic demonstrates their great potential in reducing the mobility of naphthalene and preventing abiotic loss from impacting degradation efficiency.

Materials used as a proper carriers should possess high mechanical strength, adsorption capacity, and thermal stability (Mehrotra et al., 2021). There are three commonly used carrier materials: inorganic, organic, and composite materials. Inorganic materials, such as activated carbon (AC), have the advantage of high mechanical strength, which prevents biological decomposition. However, microbial cells are easily detached due to their poor biocompatibility (Yu et al., 2020; Qi et al., 2021). Organic materials, such as alginate, exhibit good mass transfer ability. They are, however, criticized for their low mechanical strength and thermal stability, although this can be mitigated through forming calcium alginate (CA) (Gong et al., 2022). Due to these drawbacks, composite gel beads (CO) were selected and evaluated for efficacy. The main research objectives of this study are to: (1) investigate adsorption kinetics, chemical bonds, biocompatibility, and thermal stability of three carrier materials; (2) compare naphthalene degradation using free bacteria and bacteria-loaded carrier material; and (3) explore the inherent mechanisms affecting naphthalene degradation.

## 2 Materials and methods

### 2.1 Bacterial isolation and cultivation

A naphthalene-degrading bacterial strain was isolated from a petroleum-contaminated site in Yan’an, Shaanxi Province, China, and subsequently purified. The bacterial strain was identified as *Microbacterium paraoxydans* (termed ms hereafter) through DNA extraction, PCR amplification, Sanger sequencing, and alignment with the Basic Local Alignment Search Tool (BLAST) at the National Center for Biotechnology Information (NCBI) (Liu et al., 2021). The 16S rRNA sequence of the naphthalene-degrading bacteria was submitted to the NCBI database with the accession number PP702920. The degrading bacteria were then incubated in LB medium containing 10 g tryptone, 5 g yeast extract, and 10 g NaCl at 30°C and 160 rpm for 24 h. The pH of the medium was adjusted to a range of 7–7.2 using 1 mol/L NaOH and HCl solutions. Additionally, the bacterial optical density (OD) was measured to be 2.1 at 600 nm using a visible light spectrophotometer (721 G; Inesa Analytical Instrument Co., Ltd., China).

### 2.2 Bacterial-loaded carrier preparation

In this study, ms was employed not only to degrade naphthalene alone but also to be loaded onto CO. AC with an average particle size of 2–4 mm was obtained from Tianjin Damao Chemicals Reagent Partnership Enterprise. A 10% (v/v) degrading bacterial suspension was added to a mixture of 10% (m/m) AC and 4% (m/m) sodium alginate (SA) solution. This mixture was then dispensed into a 4% (m/m) CaCl_2_ solution for 3 h to prepare ms-loaded CO. Unless otherwise stated, all chemicals were of analytical purity, diluted to the necessary concentration with distilled water, and sterilized by autoclaving.

### 2.3 Batch adsorption tests

Unless stated otherwise, all adsorption tests were conducted in 50 mL sterile flasks at 30°C and 160 rpm, each containing 10 mL of naphthalene solution and 10 g/L of ms-loaded CO. The initial concentrations of naphthalene were set at 50 mg/L, 100 mg/L, and 200 mg/L. Samples used to evaluate the degradation efficiency were collected at 0.5, 1, 2, 4, 8, 12, 18, 24, 48, 72, and 96 h, respectively. The adsorption quantity *q*_*t*_ of naphthalene by CO was calculated according to Equation 1.

$$q_{t}=\frac{\left(C_{0}-C_{t}\right)V}{m}$$ (1)

where *C*_0_ and *C*_t_ are the liquid-phase concentrations of naphthalene at initial and at time t, respectively. *V* is the volume of the solution, and *m* is the mass of the carrier material used. Additionally, the results of the adsorption tests were fitted with pseudo-first-order and pseudo-second-order kinetic models, as well as the Elovich equation, to explore the pathway of naphthalene adsorption (Kumar et al., 2012; Taylor and Thon, 1952) (see Equations 2–4).

$$q_{t}=q_{e}\left(1-e^{-k_{1}t}\right)$$ (2)

$$\frac{t}{q_{t}}=\frac{1}{k_{2}q_{e}^{2}}+\frac{t}{q_{e}}$$ (3)

$$q_{t}=\frac{1}{\beta }\mathrm{Int}+\frac{1}{\beta }In\left(\alpha \beta \right)$$ (4)

where *q*_*e*_ corresponds to the adsorption quantity at equilibrium state. *k*_*1*_ and *k*_*2*_ are the pseudo-first-order rate constant and pseudo-second-order rate constant, respectively. *α* and *β* are identical to the initial sorption rate and desorption constant, respectively, related to the extent of surface coverage and activation energy for chemisorption.

### 2.4 Biodegradation tests

The mineral salt medium (MSM) (L^-1^) used for the biodegradation tests consisted of 0.6 g Na_2_HPO_4_, 0.4 g KH_2_PO_4_, 0.5 g (NH_4_)_2_SO_4_, 0.05 g CaCl_2_•2H_2_O, 0.2 g MgSO_4_•7H_2_O, and 1 mL of a trace element solution (1 g FeSO_4_•7H_2_O, 0.1 g ZnCl_2_, 0.1 g Cu(NO_3_)_2_•3H_2_O, 0.3 g MnSO_4_•H_2_O, 0.5 g NiCl_2_•6H_2_O, and 0.1 g CoCl_2_•6H_2_O per 1 L of distilled water). The tests were conducted in a thermostatic shaking incubator at 30°C and 160 rpm for 4 days. ms was inoculated into MSM with the addition of 50, 100, and 200 mg/L naphthalene at a 5% inoculation proportion (v/v). Non-inoculated sterile controls (termed CG hereafter) were also established to assess the abiotic loss of naphthalene. Samples from the inoculated tests and the non-inoculated sterile controls were taken at 0, 1, 2, 3, and 4 days to evaluate the degradation efficiency. The adsorption and biodegradation tests were conducted in triplicate to ensure repeatability.

Naphthalene, while assessing the degradation efficiency, was extracted using liquid phase ultrasonic extraction, followed by thermogravimetry analysis coupled gas chromatography-mass spectrometry (TG-GC/MS) (ISQ 7000; Thermo Fisher Scientific Inc., United States). This allowed us to identify the remaining concentration of naphthalene via the standard curve. Nitrogen served as the carrier gas in the GC/MS analysis, with a constant flow rate of 1.5 mL/min. The splitless mode was applied for sample injection at 280°C. The heating program started at 50°C and increased to 250°C at a rate of 20 C/min. The program was maintained for 1 min at 50°C and for 5 min at 250°C. The GC/MS platforms operated in electron ionization (EI) mode for mass spectral matching and metabolite annotation. The MS transfer line and ion source temperatures were set to 280°C and 300°C, respectively. The degradation efficiency can be assessed using Equation 5.

$$\text{Degradation efficiency}=\frac{C_{0}–C_{1}–C_{2}}{C_{0}}\times 100\%$$ (5)

where *C*_0_, *C*_1_, and *C*_2_ are the initial concentration of naphthalene, the concentration of naphthalene in solution after the tests, and the concentration of naphthalene on the ms-loaded CO after the tests, respectively.

### 2.5 Optical microscopy

The confocal laser scanning microscope (CLSM) offers significant advantages over conventional microscopes by rejecting light that does not originate from the focal plane, hence the term ‘confocal.’ In this study, samples that had not been exposed to naphthalene and those exposed to 100 mg/L naphthalene for 4 h, 2 days, and 4 days, respectively, were analyzed using a CLSM (FV1200; Olympus, Japan). A gel bead induced by the samples was placed on a slide, covered with a coverslip, and imaged at different magnifications to investigate the footprint of abiotic loss. The excitation wavelength and emission transmission ranges used were 530–585 nm and 615 nm, respectively.

## 3 Results and discussion

### 3.1 Adsorption kinetics

**FIGURE 1:**
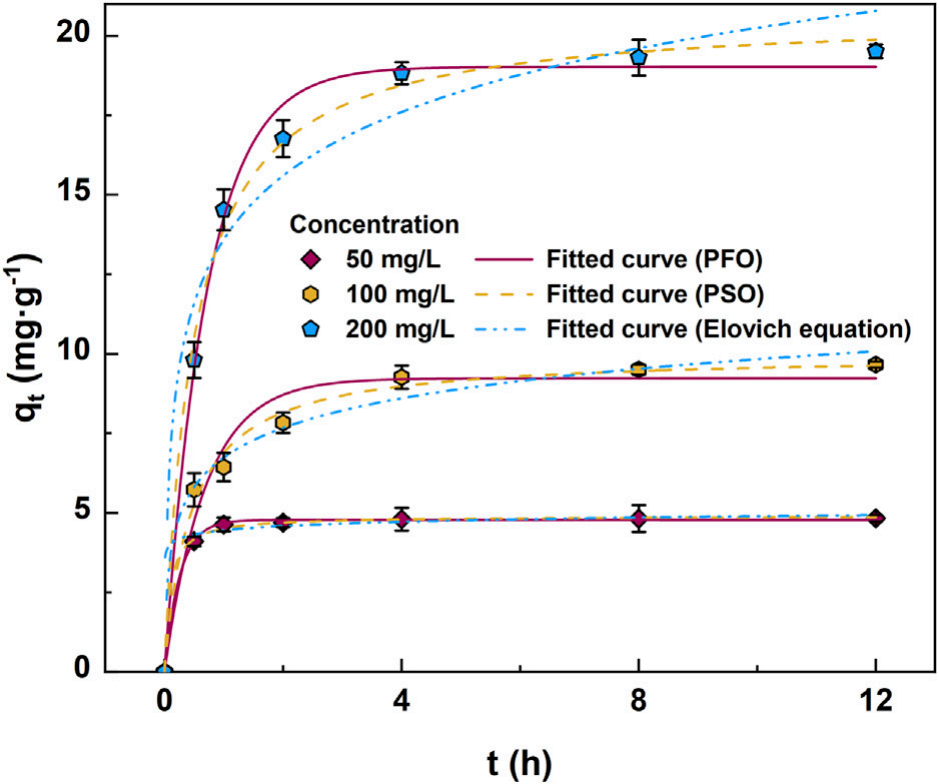
Fitting curves of adsorption kinetics for composite gel beads (CO).

The content below primarily discusses the adsorption kinetics for CO without bacterial loading, as AC and CA have not yet been selected and evaluated for efficacy. The adsorption kinetics curves and fitting parameters for CO, when exposed to 50 mg/L, 100 mg/L, and 200 mg/L naphthalene, are illustrated in Figure 1 and Table 1. The pseudo-second-order kinetic model, widely used in adsorption studies, was found to most effectively explain the adsorption kinetics, with *R* ^2^ values of 0.999, 0.992, and 0.996 for 50, 100, and 200 mg/L naphthalene, respectively. This indicated that naphthalene adsorption by CO primarily occurred via chemisorption, involving the formation of chemical bonds between the adsorbate and the adsorbent (Wang et al., 2020). The pseudo-first-order kinetic model also fit well, with *R* ^2^ values of 0.999, 0.967, and 0.994 for 50, 100, and 200 mg/L naphthalene, respectively, indicating that physisorption, involving weaker interactions such as hydrophobic interactions and Van der Waals forces, might also play a key role in the adsorption process. Additionally, the Elovich equation, used to describe adsorption kinetics on heterogeneous surfaces (Perez-Marin et al., 2007), was applied in this study. The high *R* ^2^ values of 0.993, 0.979, and 0.957 for 50, 100, and 200 mg/L naphthalene, respectively, suggested that the aromatic compounds on the AC’s surface interacted with the aromatic ring structure of naphthalene through π-π conjugation, and that Ca^2+^ in CA bound with naphthalene through Ca-π interaction. These interactions enhanced CO’s adsorption of naphthalene. In short, the pseudo-second-order kinetic model was most effective in explaining the adsorption kinetics, indicating chemisorption as the primary mechanism. The pseudo-first-order kinetic model also fit well, suggesting a role for physisorption, while the Elovich equation described the adsorption kinetics on heterogeneous surfaces, highlighting specific interactions between the adsorbent and adsorbate. The section below emphasizes the advantages of CO by comparing its chemical bonds, biocompatibility, and thermal stability with those of AC and CA.

**TABLE 1:** Summary of fitting parameters of adsorption kinetics applied to composite gel beads (CO).

Adsorption kinetic model	Parameter	Initial concentration of naphthalene (mg/L)		
		50	100	200
Pseudo-first-order	q_e_ (mg·g^-1^)	4.791	9.427	19.256
	k_1_ (h^-1^)	3.837	1.356	1.347
	*R* ^2^	0.999	0.967	0.994
Pseudo-second-order	q_e_ (mg·g^-1^)	4.875	9.962	20.381
	k_2_ (g·mg^-1^·h^-1^)	2.474	0.226	0.106
	*R* ^2^	0.999	0.992	0.996
Elovich equation	α (mg·g^-1^·h^-1^)	3.980E12	560.140	999.219
	β (g·mg^-1^)	6.919	0.912	0.440
	*R* ^2^	0.993	0.979	0.957

### 3.2 Chemical bonds and biocompatibility

**FIGURE 2:**
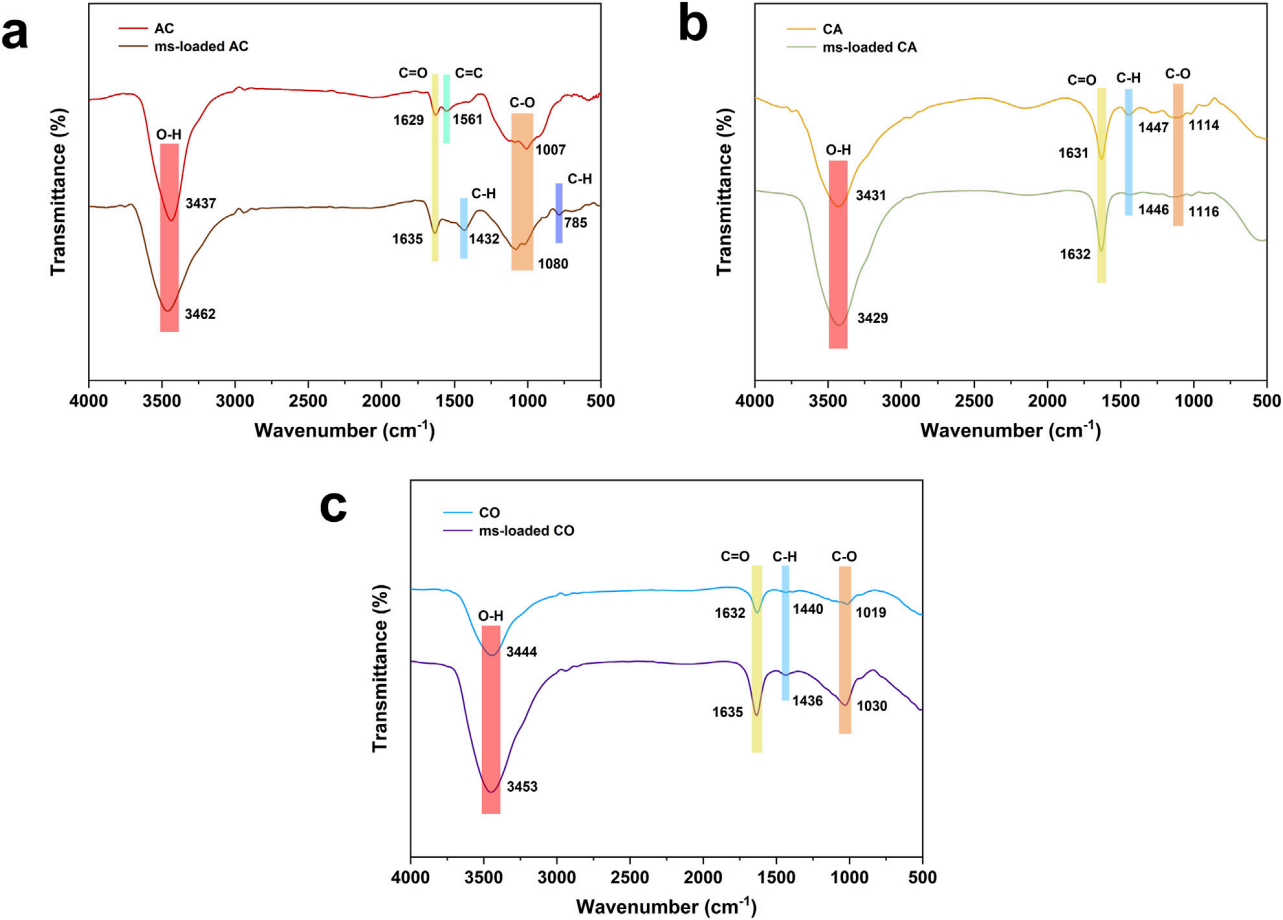
FTIR spectrum applied to free bacteria and bacteria-loaded carrier material: **(a)** activated carbon (AC) and *Microbacterium paraoxydans*-loaded activated carbon (ms-loaded AC), **(b)** calcium alginate (CA) and *Microbacterium paraoxydans*-loaded calcium alginate (ms-loaded CA), and **(c)** composite gel beads (CO) and *Microbacterium paraoxydans*-loaded composite gel beads (ms-loaded CO).

AC exhibited absorption peaks at 3,437 cm^−1^, 1,629 cm^−1^, and 1,007 cm^−1^, corresponding to the stretching vibrations of O-H, C=O, and C-O, respectively (Bao et al., 2008; Ren et al., 2022) (Figure 2). These peaks were developed due to the presence of oxygen-containing functional groups. Additionally, AC showed an absorption peak at 1,561 cm^−1^, indicating the C=C stretching vibration of aromatic compounds. This result supported the argument that aromatic compounds interacted with the aromatic ring structure of naphthalene through π-π conjugation, despite AC’s limited adsorption capacity (Xu et al., 2022). It is worth mentioning that the involvement of bacteria caused shifts in its absorption peaks (3,462 cm^−1^, 1,635 cm^−1^, and 1,080 cm^−1^) at the same chemical bonds (O-H, C=O, C-O), indicating poor biocompatibility, which led to bacteria detaching easily (Liao et al., 2022).

CA exhibited absorption peaks at 3,431 cm^−1^, 1,631 cm^−1^, 1,447 cm^−1^, and 1,114 cm^−1^, corresponding to the stretching vibrations of O-H, C=O, C-H, and C-O, respectively (Figure 2). These peaks were formed as a result of hydrophilic and hydrophobic groups (Larosa et al., 2018). The involvement of bacteria caused smaller shifts in its adsorption peaks, indicating better biological affinity and biocompatibility. While CA benefited from a three-dimensional network structure that aided naphthalene adsorption, it suffered from significant volume shrinkage when subjected to lyophilization, based on prior tests in extreme environmental settings, despite having better biocompatibility than AC.

CO exhibited absorption peaks at 3,444 cm^−1^, 1,632 cm^−1^, 1,440 cm^−1^, and 1,019 cm^−1^, which were similar to those of CA (Figure 2). Unlike AC and CA, the peaks did not show any shifts after the involvement of bacteria. CO not only addressed the limitations of both AC and CA by avoiding volume shrinkage and resolving issues related to adsorption capacity but also demonstrated the best biocompatibility among the carrier materials, as the involvement of bacteria did not cause any shifts in its adsorption peaks. CO emerged as the most effective carrier material because of its superior adsorption capacity, resistance to volume shrinkage, and excellent biocompatibility.

### 3.3 Thermal stability

**FIGURE 3:**
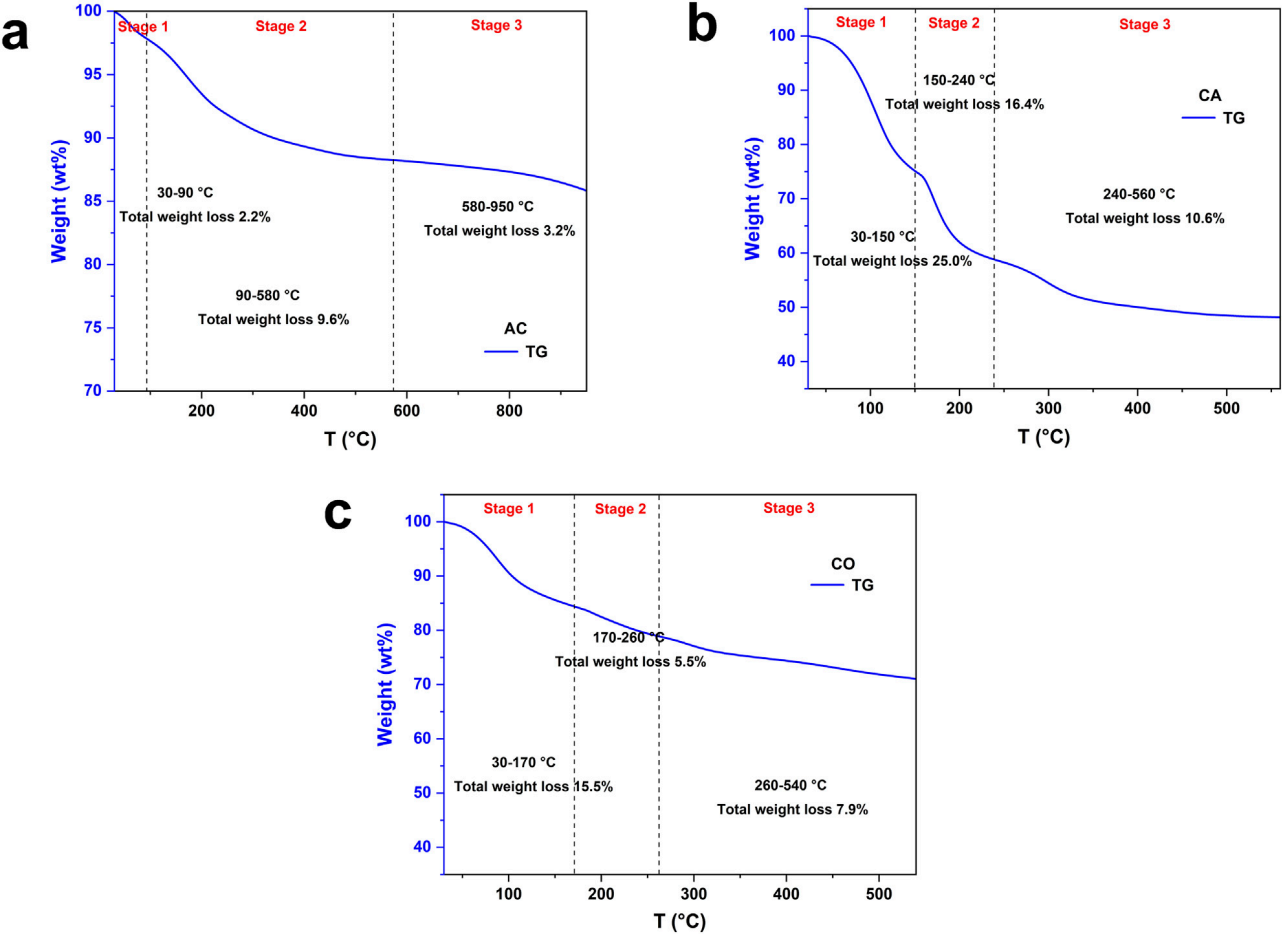
Thermogravimetric (TG) curves for: **(a)** activated carbon (AC), **(b)** calcium alginate (CA), and **(c)** composite gel beads (CO).

The weight loss of AC occurred in several stages: S1, S2, and S3 (Figure 3). The first stage (S1, 30°C–90°C) involved the evaporation of surface free water and organic matter, resulting in a low free water content after lyophilization and a weight loss of 2.2% at this stage. The second stage (S2, 90°C–580°C) was associated with the evaporation of bound water and the decomposition of oxygen-containing functional groups, leading to a weight loss of 9.6%. The third stage (S3, 580°C–950°C) featured the smallest weight loss among the stages, at 3.2%. Overall, AC demonstrated a total weight loss of 15%.

CA featured high water content even after lyophilization, as SA contained many hydrophilic groups. The first stage (S1, 30°C–150°C) was attributed to the evaporation of free water, resulting in a weight loss of 25% (Figure 3). Similar to AC, the second stage (S2, 150°C–240°C), with a weight loss of 16.4%, was due to the evaporation of bound water. Consequently, the water evaporation in the first two stages led to a significant total weight loss of 41.4%, which disrupted the three-dimensional network structure and affected its mass transfer capability. The third stage (S3, 240°C–560°C) was ascribed to the decomposition of active functional groups, such as -COOH, -OH, and the breakage of the polymer backbone, resulting in a weight loss of 10.6%. CA exhibited a total weight loss of 52%.

The first stage (S1, 30°C–170°C) of CO primarily involved the evaporation of free water, resulting in a weight loss of 15.5%, which was lower than that of CA at 25% (Figure 3). The second stage (S2, 170°C–260°C) was linked to the evaporation of bound water, with a weight loss of 5.5%. The third stage (S3, 260°C–540°C) was mainly attributed to the decomposition of oxygen-containing functional groups and the breakage of the polymer backbone, resulting in a weight loss of 7.9%. Since a portion of the CA skeleton was replaced by AC, the weight loss in the first two stages (21%) was significantly reduced compared to CA (41.4%), mitigating the structural destruction caused by water loss. Consequently, the total weight loss of CO (28.9%) was lower than that of CA (52%) during the heating process despite having the total weight loss higher than AC.

### 3.4 Degradation efficiency

**FIGURE 4:**
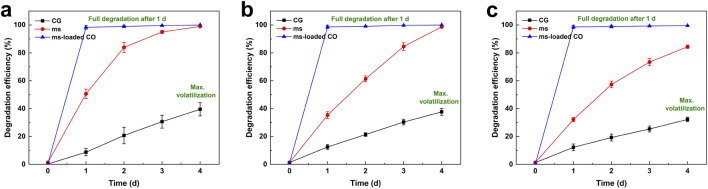
Temporal relationships of degradation efficiency observed in non-inoculated sterile controls (CG), *Microbacterium paraoxydans* (ms), and *Microbacterium paraoxydans*-loaded composite gel beads (ms-loaded CO) when exposed to **(a)** 50 mg/L, **(b)** 100 mg/L, and **(c)** 200 mg/L naphthalene, respectively.

Figure 4 compares the degradation efficiency of ms and ms-loaded CO at 50 mg/L, 100 mg/L, and 200 mg/L naphthalene. The abiotic loss, which were identical to the maximum volatilization for CG (shown by black line) on day 4, reached a significant proportion of naphthalene (135.6 mg/L, corresponding to a degradation efficiency of 32.2%), thereby highlighting the necessity of appropriate handling to mitigate severe threats to the environment and human health (Wang et al., 2023). When degrading 200 mg/L naphthalene using ms (shown by red line), 135.6 mg/L of naphthalene (32.2% efficiency) remained on day 1, and 53.2 mg/L (73.4% efficiency) remained on day 3. While the degradation efficiency reached 100% on day 4 for 50 mg/L and 100 mg/L naphthalene, 31.2 mg/L (84.4% efficiency) remained for 200 mg/L naphthalene. In contrast, when degrading 200 mg/L naphthalene using ms-loaded CO (shown by the blue line), 2.8 mg/L (98.6% efficiency) remained on day 1, and 1.6 mg/L (99.2% efficiency) remained on day 3. Similar phenomena were also observed at 50 mg/L and 100 mg/L naphthalene where a degradation efficiency of about 99% reached on day 1.

**FIGURE 5:**
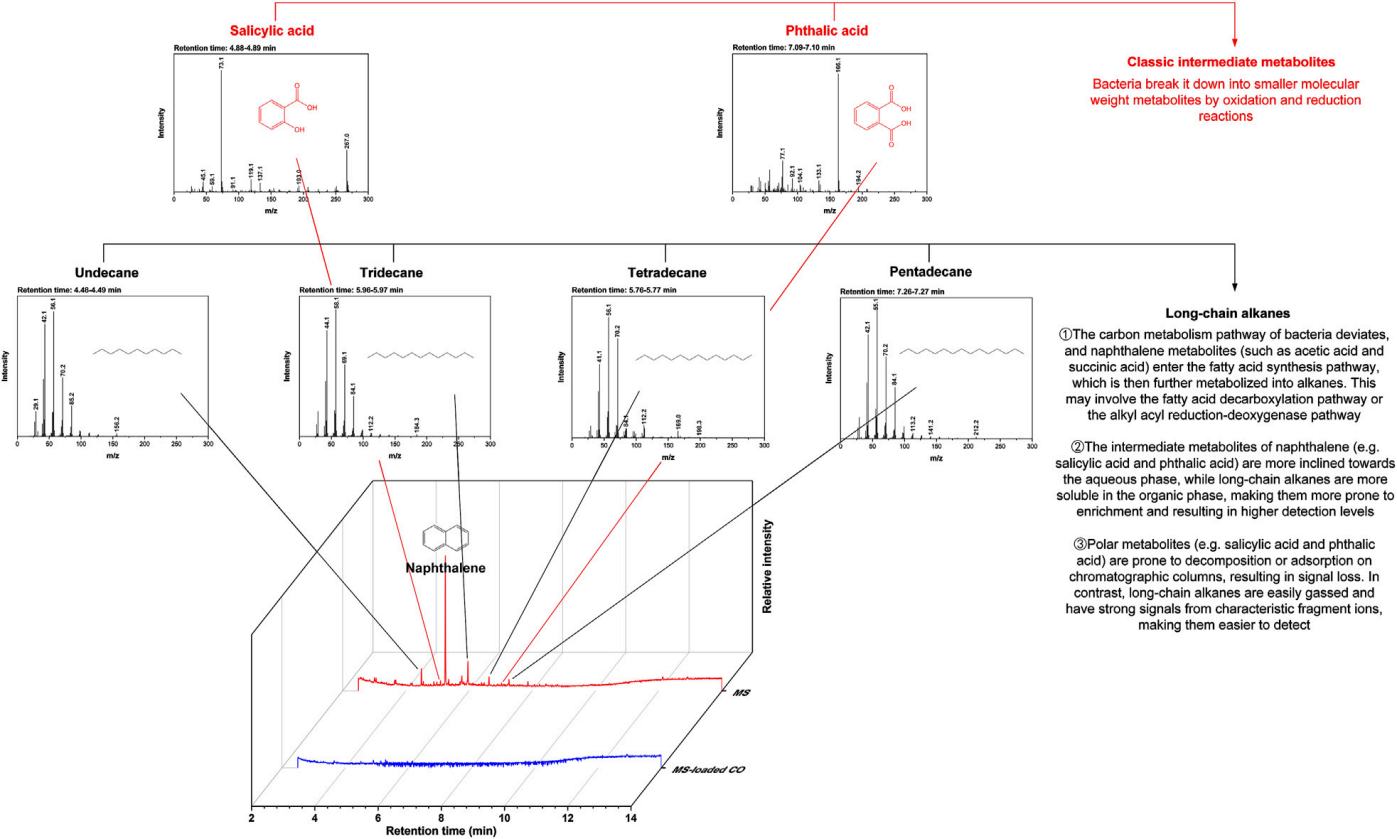
Gas chromatography/mass spectrometry (GC/MS) chromatograms.

While three metabolic pathways (including the salicylic acid pathway, the phthalic acid pathway, and the gentisic acid pathway) are often involved in naphthalene degradation, their diversity depends primarily on organisms used and cultivation conditions (Mohapatra and Phale, 2021). In this study, two metabolic pathways (the salicylic acid pathway and the phthalic acid pathway) were identified through GC/MS chromatograms (Figure 5). The degradation process started with naphthalene dioxygenase catalyzing the oxidation of naphthalene to produce cis-naphthalene dihydrodiol, which was subsequently converted by dehydrogenase into 1,2-dihydroxynaphthalene. This intermediate was cleaved into salicylic acid or phthalic acid via meta-cleavage or ortho-cleavage. The metabolic peaks confirmed the presence of salicylic acid and phthalic acid at retention times of 4.88 min and 7.09 min, respectively. The other four peaks associated with long-chain alkanes were omitted from the following discussion. The benzene ring was broken down into downstream metabolites, including pyruvic acid, acetaldehyde, acetic acid, and succinic acid. The metabolites entered the tricarboxylic acid cycle, where microorganisms used them to synthesize cellular proteins and energy, ultimately degrading them into CO_2_ and H_2_O. Overall, two metabolic pathways were identified, along with their intermediates. Table 2 presents a comparison of the degradation efficiency achieved in this study with those reported in existing literature. The existing literature showed that with immobilization carriers similar to the proposed composite beads, the degradation efficiency for diesel and pyrene removal fell within the 77%–92% range. These removals were achieved within 2–10 days, although these compounds were more stable than naphthalene and harder to break down. In contrast, a degradation efficiency of about 100% was attained on day 1, with only 2.8 mg/L remaining from the initial 200 mg/L concentration. Although the carrier materials proposed in this study have not yet been applied to diesel and pyrene removal, relevant works are ongoing, and the results will be presented in another paper.

**FIGURE 6:**
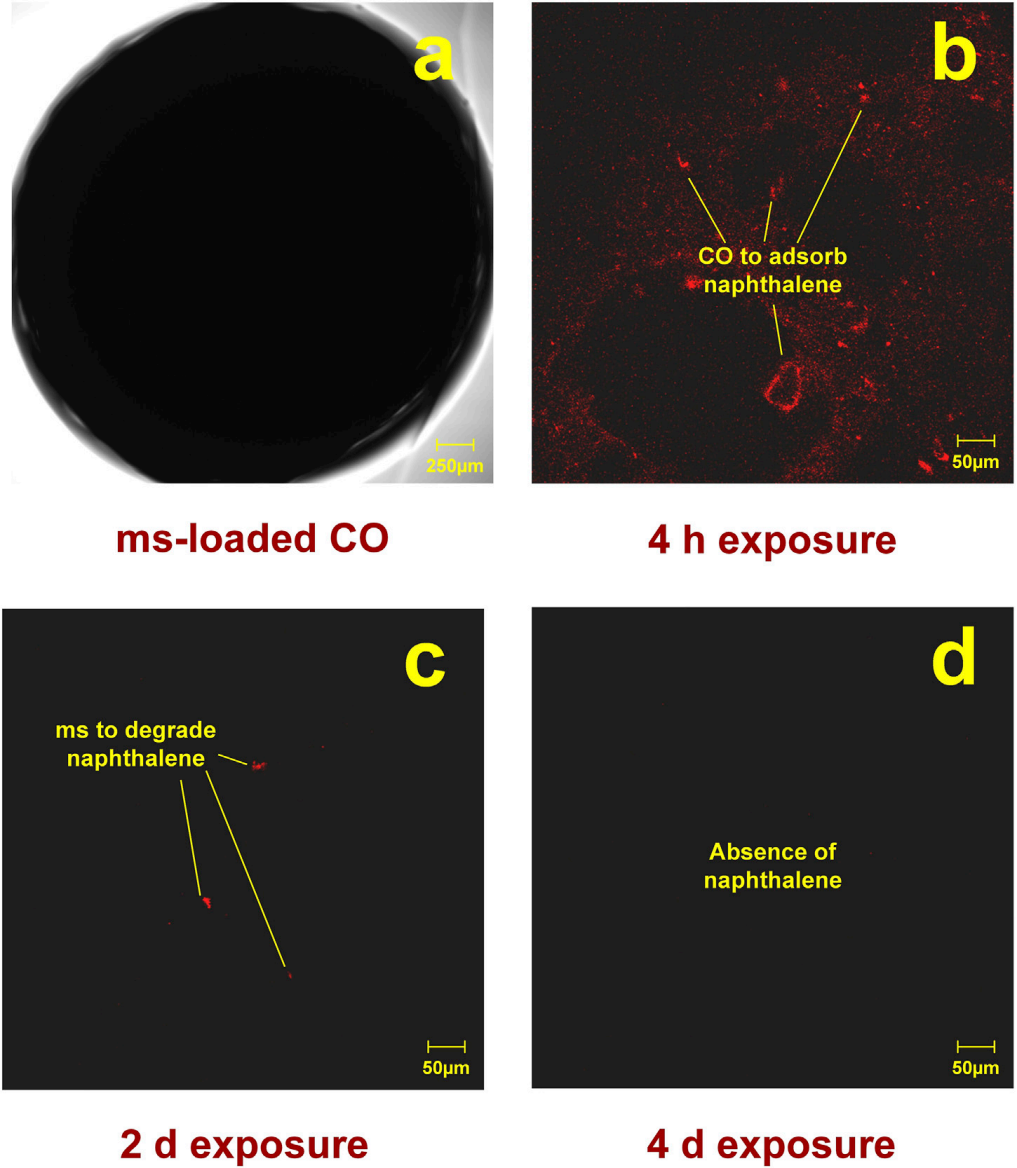
CLSM test results include: **(a)** appearance of ms-loaded CO, **(b)** initial signs of naphthalene after 4 h of exposure, **(c)** signs of naphthalene gradually disappearing after 2 days of exposure, and **(d)** absence of any signs of naphthalene after 4 days of exposure.

**TABLE 2:** Comparisons of degradation efficiency achieved in this study with those reported in the existing literature.

Contaminant	Carrier material	Treatment time	Degradation efficiency	Data source
Diesel oil	Floatable and biodegradable carrier material made by coating puffed foxtail millet (PFM) with a calcium alginate (CA)-chitosan compound membrane	2 days	Above 90%	Hou et al. (2013)
16 PAHs	Pyrene-degrading bacterial strain loaded polyvinyl alcohol-sodium alginate (PVA-SA) hydrogel beads	4 days	77%	Chen et al. (2021)
Total petroleum hydrocarbons (TPHs)	Iron-modified biochar immobilized beads (SP-FWBM)	7 days	92.4%	Chen et al. (2025)
Pyrene	Strain FA1 loaded polyvinyl alcohol (PVA)-diatomite carrier	10 days	92.8%	Xu et al. (2016)
Naphthalene	*Microbacterium paraoxydans* (ms) loaded composite gel beads	1 day	100%	This study

### 3.5 Summary

Naphthalene has a low solubility of approximately 30 mg/L in aqueous solutions. When present in high concentrations, it forms a concentrated layer on the surface, leading to a decrease in surface tension and an increase in vapor pressure. This makes naphthalene more likely to volatilize into the air, thereby posing significant health, environmental, and safety risks (Shen et al., 2007). To minimize these risks, careful handling of its volatility is required before naphthalene degradation. The present work evaluated the degradation of naphthalene using ms and ms-loaded CO. The results indicated that while ms degraded naphthalene on day 4 for 50 mg/L and 100 mg/L concentrations, 31.2 mg/L remained for the 200 mg/L concentration. In contrast, ms-loaded CO degraded most of the naphthalene on day 1, with 2.8 mg/L remaining for the 200 mg/L concentration. It was evident that high concentrations of naphthalene made it more challenging for ms to achieve effective degradation, a challenge not observed when using ms-loaded CO. Additionally, when using ms, the degradation of naphthalene was not timely, emphasizing the risk of volatilization, an issue not seen with ms-loaded CO.

The toxicity of naphthalene at high concentrations denatured ms, causing it to lose its activity. Consequently, ms was less effective in addressing both the volatilization and degradation of naphthalene. CO’s adsorption of naphthalene was mainly through chemisorption, with π-π conjugation and Ca-π interaction significantly enhancing the adsorption process. Larosa et al. (2018) characterized bare and tannase-loaded calcium alginate beads, indicating that these beads exhibited a 95% total weight loss during heating within a range of 25°C–300°C. The adsorption peaks showed significant shifts after tannase was loaded. Fontes et al. (2013) reported that antibiotic-loaded alginate-osa starch microbeads showed a 55% total weight loss while heating in a 30°C–500°C range. Similarly, distinct shifts in the adsorption peaks were observed after the antibiotic was loaded. Feng et al. (2020) developed a semi-interpenetrating network hydrogel by combining starch, alginate, and poly (N-isopropylacrylamide), indicating that this hydrogel suffered a significant weight loss of 55% during heating within a 30°C–500°C range. This combination also modified the structure of the carrier material, with significant shifts in the adsorption peaks. In contrast, after the involvement of bacteria, the composite beads reported in this study exhibited a minimal total weight loss of 28.9% when heated in the 30°C–540°C range. Additionally, the structure of this carrier material remained unchanged after the involvement of bacteria, with no shifts in the adsorption peaks, indicating superior biological affinity. These results provided compelling evidence to support the argument that ms-loaded CO featured high thermal stability and superior biocompatibility.

The adsorption of naphthalene reduced the risks of volatilization, paving the way for its degradation. The results indicated that most of the naphthalene was degraded on day 1, with only 2.8 mg/L (approximately 98.6% efficiency) remaining from the initial 200 mg/L concentration. According to the GC/MS analysis, two metabolic pathways—the salicylic acid pathway and the phthalic acid pathway—were involved in its degradation, along with their intermediates. Additionally, the CLSM tests provided evidence supporting the reduction of volatilization risks following the adsorption of naphthalene by CO. Initial signs of naphthalene were observed after 4 h of exposure, indicating that CO had begun to adsorb the compound. These signs gradually disappeared after 2 days of exposure, during which the bacteria started degrading naphthalene. After 4 days of exposure, no signs of naphthalene were detected, suggesting that the bacteria had fully degraded the compound (Figure 6). This underscored the role of CO in not only mitigating the volatilization of naphthalene but also improving degradation efficiency, expanding the potential applications of ms-loaded CO for the degradation of naphthalene in an aqueous solution.

## 4 Conclusion

This study proposed a bacteria-loaded carrier material applied to the degradation of naphthalene in an aqueous solution. Based on the results and discussion, some main conclusions can be drawn as follows.

(1) High concentrations made it more challenging for ms to achieve satisfactory degradation. Additionally, the degradation process was not timely when using ms, thereby exacerbating the risks associated with naphthalene volatilization. These were not observed when using ms-loaded CO. ms-loaded CO degraded most of the naphthalene on day 1, with 2.8 mg/L (approximately 98.6% efficiency) remaining from the initial 200 mg/L concentration.
(2) CO’s adsorption of naphthalene was primarily through chemisorption, with π-π conjugation and Ca-π interaction enhancing the adsorption process. Unlike AC and CA, the adsorption peaks of CO did not exhibit any shifts after the involvement of bacteria, indicating its superior biocompatibility despite having the second-highest thermal stability. These results supported the claim that CO was more effective in achieving effective adsorption of naphthalene, thereby minimizing the risks associated with its volatilization.
(3) Two primary metabolic pathways—the salicylic acid pathway and the phthalic acid pathway—were involved in naphthalene degradation, along with their intermediates. Additionally, no signs of naphthalene were observed in the samples from CLSM tests, indicating that ms fully degraded naphthalene after its adsorption. This study highlighted the role of CO in mitigating the volatilization of naphthalene and improving degradation efficiency.

## Data Availability

The datasets presented in this study can be found in online repositories. The names of the repository/repositories and accession number(s) can be found in the article/supplementary material.
